# Management of Hepatocellular Carcinoma: Bangladesh Perspective

**DOI:** 10.5005/jp-journals-10018-1258

**Published:** 2018-05-01

**Authors:** Sheikh M Noor-E-Alam

**Affiliations:** Department of Hepatology, Bangabandhu Sheikh Mujib Medical University, Dhaka, Bangladesh

**Keywords:** Bangladesh, Innovative cancer therapy, Liver cancer, Management.

## Abstract

Bangladesh is one of the countries facing huge burden of hepatocellular carcinoma (HCC). Hepatocellular carcinoma is the third commonest cancer in the country and it is just behind to cancer of the lung and cancer of the stomach. Hepatitis B virus (HBV) is responsible for 66% of HCC in Bangladesh. Presumptive prevalence of HBV and hepatitis C virus (HCV) may be as high as 5.4 and 0.84%, respectively, in Bangladesh, and liver diseases occupied 8 to 12% of admission in medicine wards of Public Medical College. In this mini review, I would like to highlight the impact of HBV and HCV in the development of HCC and the management of HCC from a Bangladesh perspective.

**How to cite this article:** Noor-E-Alam SM. Management of Hepatocellular Carcinoma: Bangladesh Perspective. Euroasian J Hepato-Gastroenterol 2018;8(1):52-53.

## INTRODUCTION

The whole world is facing the burden of cancer and Bangladesh is not out of the scene. Hepatocellular carcinoma is the sixth most common malignancy in the whole world and is responsible for 7% of all malignancy. Hepatitis B virus is mainly responsible for chronic hepatitis and HCC in our country. After introduction of HBV vaccine in expanded program on immunization (EPI), prevalence of HBV is progressively decreasing. By the year 2030, practical elimination of HBV from Bangladesh is our target.

## HEPATITIS B

The prevalence of HBV in Bangladesh is 5.4%. In this HBV intermediate prevalence zone, liver disease accounts for 8 to 12% of hospital admission in private medical college medicine wards. This is a huge burden for the health system in our country. The HBV vaccine is incorporated in EPI in 2003, and EPI had 93% coverage by 2010. Though at birth vaccination was not yet introduced, HBV chronic hepatitis declined 14% (76-62%) from 2007 to 2016. In addition, HBV cirrhosis and HCC declined 11% (61-50%) and 14% (66-52%) from 2007 to 2016 and 2009 to 2016 respectively. Though the HBV-induced HCC declined about 23% from 2004 to 2016, the gloomy side of liver cancer in Bangladesh is 52%, and patient presents with large space occupying lesion of about 11 to 15 cm. Moreover, 50% of the patients are in the age group of 31 to 50 years. Prevalence of HBV surface antigen (HBsAg) in <5 years old Bangladeshis is 4.2%, and 31.9% is antihepatitis B core total positive. Early horizontal transmission is the principal mode of transmission in Bangladesh.

Intervention to prevent progression of hepatic fibrosis in chronic hepatitis B is a great challenge. Future HBV therapies rely on new targets and new drugs. Targets are polymerase inhibitors, inhibition of HBsAg release, targeting covalently closed circular deoxyribonucleic acid, inhibition of nucleocapsid assembly, etc.

## NASVAC

In the recent field of HBV treatment, hepatitis B core antigen-based vaccine is a great innovation in Bangladesh. Nasal vaccine containing recombinant HBV surface and core antigens already passed phases I, II, and III clinical trials conducted in Bangladesh. NASVAC was administered in 75 patients every 2 weekly intranasally at a dose of 100 μg for five times followed by every 2 weekly administration of 100 μg intranasally plus 100 μg subcutaneously. Seventy-six patients received injection Pegylated interferon alpha-2b at a dose of 180 μg for 48 weeks. NASVAC safety was very satisfactory. At 48 weeks of end of treatment, alanine transaminase was 77% of upper limit of normal, HBeAg seroconversion was 33.3%. Most encouraging is fibrosis was regressed and >18.0 kPa was not found in any patients receiving NASVAC. NASVAC has been licensed as drug in Cuba, Nigeria, and Angola. Phase I clinical trial is going on in Japan also.

## ANTI-HCC THERAPY

Different regimes are used in HCC management, such as liver transplantation, surgical excision, local ablation [radiofrequency ablation, percutaneous ethanol injection (PEI)], anticancer drugs (sorafenib), transarterial chemoembolization (TACE), combination therapy (TACE + sorafenib). All therapies target reduction of HCC and prolongation of life. But no therapy targets pathogenesis of HCC.

## SCOPE OF IMMUNE THERAPY IN HCC

In HCC, there is decreased immune response as cancer cells are regarded as self, immune evasion by cancer cells, mutations bypass host immunity, and problem in migration of immunocytes in cancer nodules, host remain immune compromised, proper antigen is not recognized, immunocytes cannot act appropriately. New approach introduces PEI plus dendritic cells (DC) therapy, which helps in recognition of HCC antigen from cancer nodule, converting HCC antigens to danger signal for appropriate immune response. The PEI plus DC therapy reduces tumor size and improves survival in mice model. Tumor marker also declined progressively in treated HCC patients.

Bangladesh Medical and Research Council had given the ethical clearance, and the first case is expected in November 2017.

## CONCLUSION

Bangladesh is facing huge burden of liver disease patients as it is in HBV intermediate zone. After incorporating HBV vaccine in EPI, situation is changing. Trial of new drug innovation is going on in Bangladesh. Our target is free HBV Bangladesh by 2030 and free HBV HCC also.

## References

[1] Al Mahtab M (2017). Elimination of hepatitis viruses: Bangladesh scenario. Euroasian J Hepatogastroenterol.

[2] Kumagi T, Akbar SM, Horiike N, Oni M (2003). Increased survival and decreased tumor size due to intratumoral injection of ethanol followed by administration of immature dendritic cells. Int J Oncol.

[3] Kumagi T, Akbar SM, Horiike N, Kurose K, Hirooka M, Hiraoka A, Hiasa Y, Michitaka K, Onji M (2005). Administration of dendritic cells in cancer nodules in hepatocellular carcinoma. Oncol Rep.

